# Pre-treatment plasma proteomic markers associated with survival in oesophageal cancer

**DOI:** 10.1038/bjc.2012.15

**Published:** 2012-01-31

**Authors:** P Kelly, F Paulin, D Lamont, L Baker, S Clearly, D Exon, A Thompson

**Affiliations:** 1 Dundee Cancer Centre, University of Dundee, Ninewells Hospital and Medical School, Dundee DD1 9SY, UK; 2 Post-genomics and Molecular Interactions Centre, School of Life Sciences, University of Dundee, Dow Street, Dundee, UK; 3 Department of Surgical Oncology, MD Anderson Cancer Center, 1400 Holcombe Boulevard, Houston, TX 77030, USA

**Keywords:** oesophageal carcinoma, proteomics, biomarkers, survival, disease-free survival

## Abstract

**Background:**

The incidence of oesophageal adenocarcinoma is increasing worldwide but survival remains poor. Neoadjuvant chemotherapy can improve survival, but prognostic and predictive biomarkers are required. This study built upon preclinical approaches to identify prognostic plasma proteomic markers in oesophageal cancer.

**Methods:**

Plasma samples collected before and during the treatment of oesophageal cancer and non-cancer controls were analysed by surface-enhanced laser desorption/ionisation time-of-flight (SELDI-TOF) mass spectroscopy (MS). Protein peaks were identified by MS in tryptic digests of purified fractions. Associations between peak intensities obtained in the spectra and clinical endpoints (survival, disease-free survival) were tested by univariate (Fisher's exact test) and multivariate analysis (binary logistic regression).

**Results:**

Plasma protein peaks were identified that differed significantly (*P*<0.05, ANOVA) between the oesophageal cancer and control groups at baseline. Three peaks, confirmed as apolipoprotein A-I, serum amyloid A and transthyretin, in baseline (pre-treatment) samples were associated by univariate and multivariate analysis with disease-free survival and overall survival.

**Conclusion:**

Plasma proteins can be detected prior to treatment for oesophageal cancer that are associated with outcome and merit testing as prognostic and predictive markers of response to guide chemotherapy in oesophageal cancer.

The incidence of oesophageal cancer, particularly adenocarcinoma in western populations, is increasing worldwide (Botterweck *et al*, 2000; Park, 2002; Lagergren, 2005) and carries a poor prognosis, even in the minority with resectable disease (Gilbert *et al*, 2002; Munro, 2004) for whom 5 years survival ranges from 10% to 35% (Hulscher *et al*, 2002; Thompson *et al*, 2007). Trials of neoadjuvant chemotherapy and neoadjuvant chemoradiotherapy have reported mixed results ranging from no difference in curative resection or overall survival (Kelsen *et al*, 2007) to improved resection rates and survival (MRC, 2002; Geh *et al*, 2006). A systematic review of 11 randomised controlled trials demonstrated an increase in overall survival with the use of neoadjuvant chemotherapy, but statistical significance was achieved only after 5 years (Maltaner and Fenlon, 2003). Palliative chemotherapy for advanced oesophageal cancer results in the control of symptoms for 70–80% of patients with 40–50% objective response rates but only 30–40% surviving for 1 year (Gilbert *et al*, 2002).

The ability to identify prognostic biomarkers in oesophageal cancer and to determine, at an early stage, patient prognosis and/or which patients are most likely to respond to chemotherapy could prevent patients undergoing ineffective and potentially toxic treatments and allow direction of curative or palliative treatment to those most likely to benefit. Imaging techniques such as computerised tomography, magnetic resonance imaging, endoscopic ultrasound or positron emission tomography (PET) range in their effectiveness to predict response to chemotherapy (Westerterp *et al*, 2005). For example, a recent survey of the use of FDG-PET in the prediction of response to neoadjuvant chemotherapy found considerable variability in performance and concluded that the technique should not yet be used in clinical practice to guide therapy decisions (Kwee, 2010). Pathological criteria for assessment of the degree of tumour regression in the resected oesophagus using tumour regression grades may be a significant predictor of disease-free survival (Mandard *et al*, 1994) but is not an independent prognostic indicator for oesophageal adenocarcinomas (Dunne *et al*, 2001). Pathological response using modified staging criteria has been shown to predict survival following chemoradiotherapy (Swisher *et al*, 2005). In addition, pathological response to preoperative chemotherapy has been shown to improve overall survival (Kelsen *et al*, 2007). However, neither imaging techniques nor resectional pathology have to date provided robust guidance of prognosis or potential response before or during chemotherapy.

There has been growing interest in the use of proteomic methods on peripheral blood plasma to rapidly profile protein markers which link expression of the genome with disease processes (Cahill, 2001; Plebani, 2005) and to discover novel biomarkers of therapeutic response (Smith *et al*, 2006). Liquid chromatography methods such as high performance liquid chromatography and two-dimensional liquid chromatography have been increasingly employed for protein separation and mass spectroscopy (MS) techniques such as matrix-assisted laser desorption ionisation time-of-flight (MALDI-TOF) MS used to analyse the proteins (Colantonio and Chan, 2005). The technique of surface-enhanced laser desorption/ionisation time-of-flight (SELDI-TOF) MS, in which chromatographic separation is achieved on a solid surface and the proteins analysed intact (Colantonio and Chan, 2005), has been investigated to identify serum and tissue proteomic profiles that could be used in clinical practice.

One study using SELDI-TOF MS reported four peaks of undisclosed identity that differentiated between responders and non-responders in 14 patients treated with chemoradiotherapy for oesophageal squamous cell carcinoma (Hayashida *et al*, 2005) and were superior to radiological and pathological techniques in assessing response to therapy (Ota *et al*, 2007), but no comparable studies have been performed on the now more common adenocarcinoma of the oesophagus.

We have previously reported preclinical data from plasma proteomic profiling in human oesophageal cancer (murine) xenografts (Kelly *et al*, 2010). Comparison of treated and untreated xenograft animals, identified plasma proteins associated with treatment. The purpose of the current study was to develop the preclinical findings and test for clinical associations of plasma proteomic profiling in oesophageal cancer patients before treatment. We sought to test the hypothesis that the expression of circulating proteins, detected by plasma proteomic profiling, may provide clinically useful information about the outcomes for patients with oesophageal carcinoma.

## Materials and methods

### Collection of patient samples

**Figure 1:**
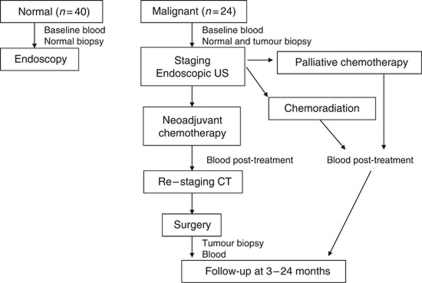
CONSORT (Schulz *et al*, 2010) diagram for the oesophageal cancer clinical study protocol. CT, computed tomography; US, ultrasound.

The study ‘Diagnostic Markers for the Detection and Monitoring of Oesophageal Disease’ was approved by the Tayside Research Ethics Board. Patients were consented on attendance for endoscopy and assigned to the normal or tumour arms of the study (Figure 1), according to the predefined inclusion and exclusion criteria such that the normal patients had no evidence of reflux disease, oesophagitis or cancer and the tumour patients were being assessed for oesophageal cancer before therapy. Baseline heparin plasma samples were collected from all study participants and submitted to the Tayside Tissue Bank for storage at −70 °C or below.

Data for each patient included dates of diagnosis, chemotherapy, surgery, recurrence, follow-ups and death (as applicable), allowing a determination to be made of survival and disease-free survival, details of chemotherapy and/or radiotherapy given, pathological response (following surgery) expressed as tumour regression grade according to the Mandard criteria (Mandard *et al*, 1994), tumour type and staging.

### Statistical considerations

To perform power calculations, data obtained in samples from five patients before and after chemotherapy (Kelly *et al*, 2010) was analysed. A total of 21 peaks were identified that differed significantly (paired *t*-test) before and after chemotherapy. Using the mean and s.d. data for each of these 21 peaks, it was determined that 20 samples would be sufficient to detect differences between these sample groups for 17 out of 21 (81%) of the peaks with a power of 70%, while 25 samples would be sufficient to detect 17 out of 21 peaks (81%) with a power of 80% and 14 out of 21 peaks (67%) with a power of 90%. These calculations are in line with the information obtained previously from the literature (Liu and Hwang, 2007). A target for recruitment was therefore 25 samples, recognising analysis on 20 patients, allowing for any necessary exclusions, could be undertaken. Sample statistics were generated to test for significant differences in peak intensities between patient groups (ANOVA).

The relationship between peak intensities, survival status and disease-free status was further analysed at various time points by univariate analysis using two-sided Fisher's exact test with Lancaster's mid-P correction (2FET) and the independent association of each variable further analysed by multivariate analysis using binary logistic regression (BLR). The statistical package used for all analyses including generation of contingency tables was MOSAIC, an internally developed statistical analysis system (implemented in Matlab, Mathworks Inc., Cambridge, UK; Version 6.5 Release 13) For all analyses the null hypothesis was rejected at an *α* level of 5% (*P*<0.05).

### Analysis of plasma samples by SELDI-TOF MS

The analysis of samples reported here was performed once all baseline samples had been collected. As such the data presented here represent an interim analysis in which not all patients were followed up for a full 2-year. All samples were analysed using a single lot of protein chips and buffers over a 3-day period. In order to minimise any differences being observed resulting from analytical effects, the samples were randomised using a random number generator (Excel spreadsheet) to determine the run order. Haemolysed samples were excluded from the analysis.

Samples from each individual were denatured in 9 M urea and tested at a final dilution of 1 : 100 on CM10 (weak cation exchanger) protein chips pre-equilibrated with 50 mM citrate, pH 4 and Q10 (strong anion exchanger) protein chips pre-equilibrated with 50 mM phosphate, pH 6. The chips were washed and sinapinic acid was added as matrix according to the manufacturer's recommended methods (Bio-Rad, Hemel Hempstead, UK), analysed using a SELDI-TOF MS PSII instrument (Bio-Rad) with an all-in-one protein standard (Ciphergen, Fremont, CA, USA) included on each run. Spectra for each individual were collected over a low molecular weight range (2000–30 000) and high molecular weight range (20 000–150 000) using fixed (optimised) laser intensity and detector sensitivity settings. Data collected from multiple points on each spot was collated into averaged spectra using the instrument software. The spectra from each experiment were normalised using the total ion current and calibrated from the all-in-one protein standard data. Peaks were detected using the instrument's biomarker software, using a peak threshold of 20%, a first pass signal/noise ratio of 5 and a second pass signal/noise ratio of 3. Sample statistics were generated for peaks identified using the biomarker software as described above. The performance of this test method was as previously described (Kelly *et al*, 2010).

### Protein identification

Protein peaks were identified by fractionation followed by MS/MS. Samples containing high-intensity peaks to be identified were fractionated using an off-gel fractionator fitted with 24-cm IPG strips according to the manufacturer's instructions (Agilent Technologies, Santa Clara, CA, USA). Selected fractions were concentrated and desalted in 10 mM HEPES using molecular weight cut-off filters (Vivaspin, Sartorius, Goettingen, Germany). The retentates were tested by SELDI MS using CM10, Q10, H50 and/or NP20 protein chips to confirm the optimal location of the peak of interest. The desalted fractions were reduced in 10 mM DTT (10 min incubation at 70 °C), alkylated in 50 mM iodoacetamide (30 min incubation at room temperature) and loaded onto nu-PAGE 10–20% Tricine, 10% or 12% Bis-Tris gels (Invitrogen, Carlsbad, CA, USA) with MES running buffer and run with SeeBlue Plus 2 prestained standards (Invitrogen). Electrophoresis was performed at 125 V for 70–90 min (tricine gels) or 200 V for 35–40 min (Bis-Tris gels). Gels were fixed with 50% methanol/10% acetic acid and staining was performed with Coomassie G-250 colloidal stain (Invitrogen) for 3–12 h and destained with deionised water overnight. Selected protein bands were excised from gels using a Harris Unicore 1-mm cutter (Sigma-Aldrich, Dorset, UK). Proteins were passively eluted from the gel pieces to confirm the presence of the peaks of interest by SELDI MS using CM10, Q10, H50 and/or NP20 protein chips. Matched gel pieces were processed and in-gel digested with Trypsin (Roche, Welwyn Garden City, UK) and an aliquot of the digest analysed by nLC-MS-MS using a 4000 QTRAP (Applied Biosystems, Carlsbad, CA, USA). The tandem MS data generated from the observed peptides were analysed and identified using the Mascot search engine (http://www.matrixscience.com) against the IPI human database. Only peptides that had ion scores above the significance threshold were reported and grouped into their respective protein identifications, with MOWSE scores indicating probability of correct identification. Peptide sequences obtained were mapped on the known protein sequence to determine the percentage of the sequence covered.

## Results

In all, 24 subjects with oesophageal cancer and 40 non-cancer patients were enrolled in the study. Among the 24 oesophageal cancer patients (Table 1), 20 had adenocarcinomas, 1 squamous cell carcinoma, 2 poorly differentiated carcinomas and 1 severe dysplasia. Eleven (all adenocarcinoma) patients received neoadjuvant chemotherapy followed by surgery, eight patients received palliative chemotherapy and two received chemoradiation. For the 11 adenocarcinoma patients who received neoadjuvant chemotherapy, the pathological response (tumour regression grade, TRG) was scored as TRG4 (residual cancer outgrowing fibrosis) in 4 cases and TRG5 (absence of regressive change) in 7 cases (Mandard *et al*, 1994). At the time of this interim analysis all surviving patients had been followed up for at least 12 months. Overall survival in the 24 oesophageal cancer patients ranged from 2 months to >24 months from the date of diagnosis, median survival was 16.9 months (median survival 17.5 months). Of the 13 patients who had died to date at the time of analysis, mean survival was 8.7 months (median 9.3 months). Survival in the neoadjuvant chemotherapy group ranged from 8 months to greater than 12 months. Given the survival data, it was deemed sufficient data had been generated to perform the analysis at this stage rather than waiting for all surviving patients to be followed up to 24 months.

The 40 non-cancer patients (Table 2) had a mean age of 52.5 years (median 55, range 23–79) and 13 out of 40 (32.5%) were male, whereas the oesophageal cancer group (Table 1) had a mean age of 59.5 (median 59, range 45–74) and 20 out of 24 (83.3%) were male. Hence, although any differences observed between the two groups are unlikely to be affected by the similar age ranges, differences due to gender might be observed. All but one case control and all the oesophageal cancer cases were Caucasian.

**Figure 2:**
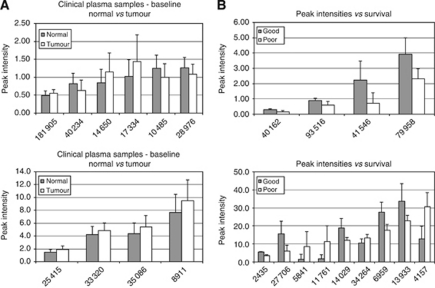
Peak intensity data at baseline for (**A**) oesophageal cancer patients *vs* case controls and (**B**) according to survival. Sample group statistics (mean and s.d.) obtained for (**A**) plasma peaks (*m*/*z*) differing significantly (*P*<0.05, appropriate parametric or non-parametric test) between case controls (normal, *n*=36) and oesophageal cancer (tumour, *n*=21) patients at baseline or (**B**) according to survival from date of diagnosis, between the greater than 11 months group (good, *n*=5) and the <7 months group (poor, *n*=5).

**Figure 3:**
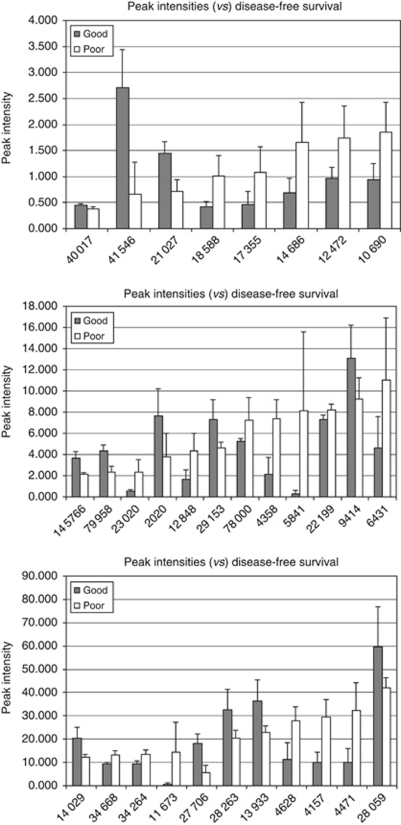
Peak intensity data at baseline according to disease-free survival. Sample group statistics (mean and s.d.) obtained for plasma peaks (*m*/*z*) differing significantly (*P*<0.05 appropriate parametric or non-parametric test), according to disease-free survival from date of diagnosis, between the >11 months group (good, *n*=4) and the <7 months group (poor, *n*=6).

SELDI-TOF MS spectra were obtained in plasma samples from the normal and malignant arms of the study at baseline, together with the plasma samples collected at specified time points (Figure 1). The spectra were analysed to detect statistically significant differences (ANOVA *P*<0.05) in peak intensities between the sample groups. Peaks that differed significantly at baseline between the non-cancer patients (*n*=36) and oesophageal cancer patients (*n*=21) are shown in Figure 2. Peaks that differed significantly between normal men and women were excluded from this analysis to correct for any gender bias between the normal and malignant groups. Protein peaks were detected that differed significantly at baseline between a group of five patients who survived for >11 months (good survivors) and a group of five patients who survived for <7 months (poor survivors, Figure 2B) or between good and poor disease-free survivors (Figure 3).

To further test the hypothesis that markers for survival and disease-free survival had been detected, statistical analysis was performed on the whole oesophageal cancer cohort after exclusion of the hyperplasia patient and three haemolysed samples (20 patients remaining). Peak intensity data generated using this dataset were assessed using histograms and dot plots to determine a cut-off and the results for each sample scored as high or low (above or below the cut-off). The relationship between a high or low baseline result and survival or disease-free survival was tested by univariate analysis using Fisher's exact test (with Lancaster's mid-point correction) and confirmed an association (*P*<0.05) between peak intensity and survival or disease-free survival (by month) for several peaks at three or more time points.

To further test the associations detected by univariate analysis, multivariate analysis was performed (BLR). From the univariate analysis, five peaks were identified that gave identical responses (all five were high in the same patients and all five were low in the same patients). These were peaks at (*m*/*z*) 27 706 and as well as peaks at (*m*/*z*) 34 264, 41 546 and 79 958. In addition, a peak at (*m*/*z*) 23 020 gave an exactly opposite response to these five markers. It was necessary to exclude all but one of these (*m*/*z* 79 958 retained) in order to perform multivariate analysis. Results obtained for the (*m*/*z*) 79 958 peak apply equally to the other four peaks and inversely for (*m*/*z*) 23 020. A further two peaks at (*m*/*z*) 5841 and 11 670 (both from the low molecular weight analysis of the CM10 chip) gave nearly identical results to the peak at (*m*/*z*) 23 020 and were similarly excluded.

Multivariate analysis was performed on all remaining peaks, to test for associations with disease-free survival at months 4 and 8, and survival at months 8 and 12. These time points were selected as optimal in terms of their bimodal discrimination for clinical survival in the cancer study population, consistent with probability values obtained for all the markers by Fisher's exact test.

**Figure 4:**
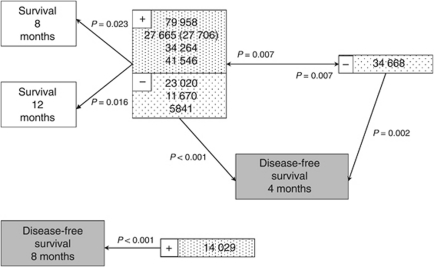
Multivariate analysis, associations with survival and disease-free survival. Associations between peaks (*m*/*z*) intensities and survival or disease-free survival determined by BLR. Direct lines indicate statistically significant independent associations (*P*-values shown). Positive (+) and negative (−) associations are depicted.

Two peaks at (*m*/*z*) 79 958 and 34 668 were found to be significantly associated (*P*<0.05) with each other and with survival at 8 and 12 months. The peak at (*m*/*z*) 79 958 was independently associated with survival, whereas the peak at (*m*/*z*) 34 668 was not. The same two peaks were both found to be independently associated with disease-free survival at 4 months. In addition, the peak at 14 029 was found to be independently associated with disease-free survival at 8 months. By their identical responses, the findings for the peak at (*m*/*z*) 79 958, also apply to the peaks at (*m*/*z*) 27 765 (or 27 706), 34 264 and 41 546, and apply negatively to the peaks at (*m*/*z*) 23 020, 11 670 and 5841 (Figure 4).

Two groups of markers were detected that were associated with each other:

1. Peaks at (*m*/*z*) 12 848, 14 686, 10 690 and 18 588. None of these were associated with survival or disease-free survival.
2. Peaks at (*m*/*z*) 4471, 28 060, 28 263, 13 933, 14 029 and 4157. This grouping includes the peak at (*m*/*z*) 14 029 that was independently associated with disease-free survival at 8 months.

From peptide fingerprinting analysis of fractionated samples, three of the protein peaks that were independently associated with survival and/or disease-free survival were identified at the appropriate expected molecular weights as serum amyloid A (*m*/*z* 11 670), transthyretin (*m*/*z* 14 029) and apolipoprotein A-I (*m*/*z* 27 665) with high Mowse probability scores (1131 or greater), multiple peptides detected (37 or more) and high sequence coverage (42% or higher).

## Discussion

This proteomic profiling study sought pre-treatment plasma proteins associated with better disease-free or overall survival following therapy for oesophageal cancer, informed by preclinical findings from a murine oesophageal cancer xenograft model.

Significant differences were observed in baseline plasma samples between the non-cancer and oesophageal cancer groups for a total of 10 protein peaks.

Univariate analysis demonstrated associations between the baseline peak intensity data for 9 protein peaks significant for survival and 14 peaks significant for disease-free survival at multiple time points (1–12 months following diagnosis). Multivariate analysis demonstrated that four of the peaks (*m*/*z* 79 958, 27 665, 34 264 and 41 546) were positively associated and three peaks (*m*/*z* 23 020, 11 670 and 5841) negatively associated independently with survival at 8 and 12 months. These peaks and a further peak (*m*/*z* 34 668) were also independently associated with disease-free survival at 4 months, and a further peak (*m*/*z* 14 029) was independently associated with disease-free survival at 8 months.

Included among the peaks that were associated with survival or disease-free survival, both by univariate analysis and multivariate analysis are three peaks identified as SAA (*m*/*z* 11 670), transthyretin (*m*/*z* 14 029) and apolipoprotein A-I (*m*/*z* 27 665). We previously reported that these three proteins were associated with response to chemotherapy treatment in a mouse xenograft model (Kelly *et al*, 2010). The present study therefore confirms that these three plasma proteins are of interest in patients with oesophageal cancer and are associated with disease-free survival or overall survival. Also included among these peaks were another five peaks (*m*/*z* 79 958, 34 264, 41 546, 23 030 and 5841), not predicted by the murine work, the identities of which are unknown.

The three peaks for which proteins were identified, Serum Amyloid A, transthyretin and apolipoprotein A-I are consistent with data from a variety of other cancers. As markers of inflammation and acute phase responses (Malmendier *et al*, 1988; Raynes *et al*, 1991; Juan *et al*, 2004; Malle *et al*, 2009) they may be non-specific markers for oesophageal cancer as individual proteins, confounded by inflammatory processes that occur in other disease states (Chechlinska *et al*, 2010). However, their detection may result from the known involvement of inflammatory mechanisms in the development of oesophageal cancer (Abdel-Latif *et al*, 2009). Indeed there is now good evidence that markers of systemic inflammatory response are prognostic cancer biomarkers, particularly in colorectal, gastro-oesophageal and renal cancers (Roxburgh and McMillan, 2010), and a prognostic score based on serum c-reactive protein and albumin (the Glasgow Prognostic Score) has been proposed for oesophageal cancer (Vashist *et al*, 2011). Therefore, the combination of changes observed in plasma for the markers observed in the present study merits testing as a prognostic signature for oesophageal cancer, a hypothesis that would require further validation.

A similar study performed by SELDI-TOF MS in patients with oesophageal squamous cell carcinomas proposed four unidentified serum markers (*m*/*z* 7420, 9112, 17 123 and 12 867) associated with chemoradiotherapy response in 14 out of 15 patients (Hayashida *et al*, 2005) as the only significant association with outcome by multivariate analysis (Ota *et al*, 2007). The current study, performed with plasma samples predominantly in patients with adenocarcinomas, did not confirm this previously reported serum panel. One peak with a very similar *m*/*z* ratio of 12 848 was detected but not found to be associated with survival or disease-free survival. The different markers detected in these two studies may reflect differences resulting from sample collection (the use of serum *vs* plasma) and processing, including differences in selection of chip surfaces and/or buffer conditions, or differences in the patient population (both geographic and squamous cell *vs* adenocarcinoma), reflecting the differences seen in the preclinical models of OE21 (squamous cell carcinoma) and OE19 (adenocarcinoma) treatment markers (Kelly *et al*, 2010). Alternatively such differences may simply reflect the small population sizes in the two studies pointing to the need for further validation cohorts.

Much has been made in the literature (Kerr and Midgley, 2010) about the plethora of candidate markers that have been proposed in the cancer field, particularly in the case of proteomics studies where underpowered studies have resulted in marker profiles which cannot subsequently be validated (McLerran *et al*, 2008). The clinical study described here has extended preclinical xenograft data (Kelly *et al*, 2010) and demonstrated associations between baseline (pre-treatment) plasma proteins and oesophageal cancer survival. The variety of therapeutic approaches, small cohort size and poor responses to therapy do not allow for testing the predictive value of the plasma markers in response to therapy, but warrant further clinical investigation involving a larger patient cohort, both to independently verify the prognostic findings and to further elucidate the predictive clinical utility of these markers.

## Figures and Tables

**Table 1:** Summary of oesophageal cancer patients

**Study number**	**Age at diagnosis (years)**	**Gender**	**Ethnic origin**	**Tumour type**	**Chemotherapy**	**Drug regime**	**Further treatment**	**Tumour regression grade**
T001	66	Male	White	Adenocarcinoma	Neoadjuvant	CF	Surgery	TRG5
T002	58	Male	White	Adenocarcinoma	Neoadjuvant	CF	Surgery	TRG5
T003	62	Male	White	Adenocarcinoma	Neoadjuvant	CF	Surgery	TRG5
T004	62	Male	White	Adenocarcinoma	Neoadjuvant	CF	Surgery	TRG4
T005	57	Male	White	Adenocarcinoma	Neoadjuvant	ECF	Surgery	TRG5
T006	58	Male	White	Adenocarcinoma	Neoadjuvant	CF	Surgery	TRG4
T007	51	Male	White	Adenocarcinoma	Neoadjuvant	CF	Surgery	TRG4
T008	58	Male	White	Adenocarcinoma	Chemoradiation	CF	Stent	N/A
T009	72	Male	White	Squamous cell carcinoma	Palliative	ECF	Stent	N/A
T010	68	Male	White	Poorly differentiated carcinoma	Palliative	ECF	None	N/A
T011	49	Male	White	Poorly differentiated carcinoma	Palliative	ECF	None	N/A
T012	52	Male	White	Adenocarcinoma	Palliative	ECF	Stent/radiation	N/A
T013	60	Male	White	Dysplasia	None	None	None	N/A
T014	45	Female	White	Adenocarcinoma	Palliative	ECF	Radiation	N/A
T015	60	Male	White	Adenocarcinoma	Neoadjuvant	CF	Surgery& radiation	TRG5
T016	54	Male	White	Adenocarcinoma	Neoadjuvant	ECF	Surgery	TRG5
T017	68	Male	White	Adenocarcinoma	None	None	Radiation	N/A
T018	64	Male	White	Adenocarcinoma	None	None	None	N/A
T019	51	Male	White	Adenocarcinoma	Palliative	ECF	None	N/A
T020	74	Female	White	Adenocarcinoma	Palliative	ECaF	None	N/A
T021	49	Male	White	Adenocarcinoma	Neoadjuvant	CF	Surgery	TRG4
T022	71	Female	White	Adenocarcinoma	Palliative	MF	None	N/A
T023	58	Female	White	Adenocarcinoma	Neoadjuvant	CF	Surgery	TRG5
T024	64	Male	White	Adenocarcinoma	Chemoradiation	F	Radiation	N/A

Abbreviations: C=cisplatin; Ca=carboplatin; E=epirubicin; F=5-fluorouracil; M=mitomycin c.

**Table 2:** Summary of case controls

**Study number**	**Age at recruitment (years)**	**Gender**	**Ethnic origin**	**Indication for endoscopy**
N001	54	Female	White	Microcytic anaemia
N002	26	Male	White	Coeliac screening
N003	23	Female	White	Anaemia
N004	53	Female	White	Anaemia
N005	54	Female	White	Anaemia
N006	46	Female	White	Diarrhoea
N007	74	Male	White	Anaemia
N008	34	Male	White	Dyspepsia
N009	44	Female	White	Epigastric pain
N010	63	Female	White	Epigastric pain
N011	77	Female	White	Dyspepsia, dilated pancreatic duct
N012	53	Male	White	Epigastric Pain
N013	69	Female	White	Dyspepsia
N014	59	Female	White	Dyspepsia, previous salmonella
N015	70	Male	White	Maleana
N016	36	Female	White	Abdominal pain
N017	46	Female	White	Dyspepsia
N018	58	Female	White	Microcytic anaemia
N019	41	Male	White	Epigastric Pain
N020	58	Male	White	Nausea and vomiting
N021	24	Female	White	Coeliac screening
N022	60	Female	White	Iron deficiency anaemia
N023	57	Female	White	Microcytic anaemia
N024	39	Male	White	Dyspepsia
N025	64	Male	White	Dyspepsia
N026	59	Female	White	Iron deficiency anaemia
N027	73	Female	White	Altered bowel habit and anaemia
N028	46	Female	White	Anaemia
N029	49	Female	White	Abdominal pain and weight loss
N030	58	Female	White	Weight loss
N031	79	Male	White	Anaemia
N032	69	Male	White	Anaemia
N033	23	Female	White	Dyspepsia
N034	24	Male	Asian	Weight loss
N035	54	Female	White	Dyspepsia
N036	71	Female	White	Weight loss
N037	65	Female	White	Dyspepsia and weight loss
N038	56	Female	White	Weight loss and vomiting
N039	32	Male	White	Abdominal pain and possible maleana
N040	63	Female	White	Weight loss
